# Gelatine Film Incorporated with *Clitoria ternatea*-Derived Anthocyanin Microcapsules, A Food Packaging Material Effective Against Foodborne Pathogens

**DOI:** 10.17113/ftb.59.04.21.7069

**Published:** 2021-12

**Authors:** Chean Ring Leong, Nurul Shahida Daud, Woei Yenn Tong, See Yuan Cheng, Wen Nee Tan, Nurhanis Syafiqah Hamin, Khairul Faizal Pa'ee

**Affiliations:** 1 Universiti Kuala Lumpur, Malaysian Institute of Chemical and Bioengineering Technology, Lot 1988 Kawasan Perindustrian Bandar Vendor, Taboh Naning, 78000 Alor Gajah, Melaka, Malaysia; 2 Faculty of Mechanical Engineering, Universiti Teknikal Malaysia Melaka, Hang Tuah Jaya, 76100, Durian Tunggal, Melaka, Malaysia; 3 School of Distance Education, Universiti Sains Malaysia, Minden, Penang, Malaysia

**Keywords:** gelatine film, food packaging, anthocyanins, natural antimicrobial agent, foodborne pathogens, *Clitoria ternatea*

## Abstract

**Research background:**

Microbial contamination of food products is one of the significant causes of food spoilage and foodborne illnesses. The use of active packaging films incorporated with antimicrobial agents can be a measure to improve food quality and extend shelf life. Nevertheless, antimicrobial agents such as silver, copper, titanium and zinc in the packaging films have raised concerns among consumers due to toxicity issues.

**Experimental approach:**

The current study aims to develop biodegradable gelatine-based edible films incorporated with microcapsules of *Clitoria ternatea*-derived anthocyanins as a natural antimicrobial agent. The impact of incorporation of microcapsules with anthocyanins on the morphology, thermal, mechanical, water vapour barrier and physicochemical properties of the gelatine films was evaluated in this study. The effectiveness of the developed films against foodborne pathogens and their application for perishable food protection were also investigated.

**Results and conclusions:**

The results show that incorporating anthocyanin microcapsules enhances the gelatine film physical and mechanical properties by increasing the thickness, tensile strength, Young's modulus and elongation at break of the films. Scanning electronic microscopy analysis revealed that the film surface morphology with anthocyanin microcapsules had a homogeneous and smooth surface texture compared to the control. The thermogravimetric analysis also showed a slight improvement in the thermal properties of the developed films. Agar well diffusion assay revealed that the developed films exhibit significant inhibition against a broad-spectrum of bacteria. Furthermore, the films composed of gelatine with anthocyanin microcapsules significantly reduced the total viable count of microorganisms in the bean curd during storage for 12 days compared with the control films.

**Novelty and scientific contribution:**

Increasing global awareness of healthy and safe food with minimal synthetic ingredients as preservatives has sparked the search for the use of antimicrobial agents of natural origins in active food packaging material. In this study, a safe and effective active packaging film was developed using an environmentally friendly biopolymer, gelatine film incorporated with microcapsules of *Clitoria ternatea*-derived anthocyanins as a natural antimicrobial agent. This study demonstrated that such a method is not only able to improve the film physical properties but can also significantly prolong the shelf life of food products by protecting them from microbial spoilage.

## INTRODUCTION

Food spoilage is a complex process resulting from the biochemical changes caused by microbial population, which leads to the loss of nutritional value, texture and flavour of the food (*1*). Despite the improvement of food processing and production with modern technology, microbial food spoilage is still a major global issue that causes excessive food waste. The Food and Agriculture Organization of the United Nations reported the waste of 1.3 billion tonnes of food per year due to food spoilage, which constitutes an enormous financial burden of 750 billion USD (*2*). Furthermore, ingestion of the microorganisms or the microbial contaminants is linked to foodborne illnesses, including common symptoms such as diarrhoea, nausea and vomiting (*3*). Foodborne illnesses have accounted for 3% of mortality worldwide and costs of more than 15.6 billion USD annually (*4*).

Food packaging is used to improve the safety and prolong the shelf life of food products by postponing microbial spoilage. Non-biodegradable polymers like plastics are commonly used as food packaging material. Nevertheless, petroleum-based food packaging materials are being phased out in favour of biopolymer-based biodegradable packaging due to environmental concerns. On the contrary, upon disposal, biodegradable packaging materials including polysaccharides, protein or lipids will be broken down by microorganisms (bacteria, fungi and algae) through composting process to carbon dioxide, water, methane and biomass (*5*). Soya protein, maize zein, sodium caseinate, wheat gluten, pea proteins, sunflower protein and gelatine have all been utilized to make biodegradable films (*6*). Among these biodegradable protein films, gelatine-based films have a significant potential for commercial use as food packaging films due to their associated and unique features. Gelatine is a natural water-soluble protein characterized by the absence of a noticeable odour and the random configuration of polypeptide chains in an aqueous solution (*7*). It is obtained from the partial hydrolysis of collagen, a fibrous protein mainly found in certain parts of vertebrate and invertebrate animals as bones, skins, connective tissues and tendons. Film-forming properties of gelatine have prompted considerable interest as edible food packaging films, which served as an excellent alternative to conventional plastic food packaging that causes various environmental issues.

Active food packaging films incorporating synthetic or natural antimicrobial agents are among the recent solutions in preventing microbial spoilage. Various agents, including metals, salts, organic acids and chitosan, have been studied in antimicrobial packaging with varying degrees of success (*8*, *9*). Among all, the use of inorganic and metal nanoparticles such as Ag, TiO_2_, ZnO, Cu_2_O and CuO in food packaging has been reported with fairly good antimicrobial activity. However, concerns have been raised over the risks associated with the potential ingestion of Ag ions that can migrate into food and drinks. Lansdown (*10*) has reported that chronic ingestion or inhalation of silver can lead to the deposition of silver metal/silver sulfide particles in the skin (argyria), eye (argyrosis) and other organs. Thus, there is a growing interest in the development of antimicrobial packaging materials with natural antimicrobial agents. Numerous research studies have documented the effective production of smart food packaging materials employing natural components such as citric acid, pomegranate extract, apple peel extract and purple cabbage extract in recent years (*11*, *12*). The use of these pigments or compounds not only improves the antioxidant and antibacterial characteristics of the food packing materials but also allows colour changes to occur, which are related to pH shifts during food deterioration (*13*, *14*).

Anthocyanins are pigments found in various flowers, vegetables, fruits and berries (*15*). Traditionally, they have been used as a natural food colourant. These natural pigments are bioactive substances reported with strong oxidation resistance and therapeutic benefits against inflammation and infections (*16*, *17*). In recent years, anthocyanins have been gaining attention as food additives in the food industries (*18*). *Clitoria ternatea*, commonly known as butterfly pea, belongs to the *Fabaceae* family and is native to Asian countries (*19*). *C. ternatea* is a flower crop with edible flowers used as a natural colourant for various local delicacies and served as tea in South East Asia. It is rich in pigments, mostly anthocyanins (*20*). Our previous study has shown that *C. ternatea*-derived anthocyanins exhibited antimicrobial effects against a broad spectrum of foodborne pathogenic bacteria (*21*). However, anthocyanins are sensitive to the environment conditions and are easily degraded, resulting in their reduced bioavailability (*22*).

In the present study, we have developed the gelatine-based film incorporated with microcapsules of *C. ternatea*-derived anthocyanins as natural antimicrobial agents for application in the food packaging industry. The effect of anthocyanin microcapsule incorporation on the film physical properties and the gelatine film antimicrobial properties against foodborne pathogens were evaluated. The antimicrobial efficiency of the developed food packaging material was also investigated using a food model.

## MATERIALS AND METHODS

### Plant material

Plant samples of *Clitoria ternatea* were gathered in Bandaraya Melaka, Malacca, Malaysia (N2°12'3" E102°14'21"). Botanists from Universiti Kuala Lumpur had verified the material. Fungicides and pesticides are not used in the crop area. Only flowers with no evident signs of disease were collected by hand for sampling. The plant material was collected and placed in resealable plastic bags for processing within 24 h. The samples were cleaned in the lab under running tap water. The flowers were dried at 60 °C in a convection drying oven (Vac Oven 19.8L; Thermo Scientific, Waltham, MA, USA) until they reached a constant mass. A food blender was used to grind the dried flower (800G/S; MRC Laboratory Instruments, Harlow, UK). The powdered ingredients were kept in a desiccator until they were needed again.

### Anthocyanin extraction from C. ternatea

Anthocyanins were extracted from *C. ternatea* according to a previously reported method (*21*). Briefly, the *C. ternatea* flowers were dried at 60 °C until constant mass and ground into fine particles. The anthocyanin content was extracted by soaking the powdered plant material in acidified ethanol (pH=4.5) for three days at a ratio of 1:20 (*m*/*V*). The extract was then dried under reduced pressure by using a rotary evaporator (Rotavapor R-100; Buchi, Flawil, Switzerland) at 50 °C. The qualitative anthocyanin analysis was conducted by adding to the extract equal volume of 2 M hydrochloric acid and ammonia solution. The absorbance of the blue-violet solution measured at 520 nm using a spectrophotometer (UV-1280; Shimadzu, Tokyo, Japan) indicates a positive result for anthocyanins.

### Preparation of anthocyanin microcapsule suspension

The microcapsules were prepared with the hot homogenization technique with slight modifications (*23*). A volume of 100 mL of 2% of nonionic surfactant (pluronic F127, Sigma-Aldrich, Merck, St Louis, MO, USA) solution was prepared in distilled water in an ice bath. Then, 200 mL of anthocyanin extract were added. The solution was then mixed with a homogenizer (Heidolph, Schwabach, Germany) at 10 000 rpm for 2 min. To prepare the solid phase, 1% dextran (Sigma-Aldrich, Merck) solution was prepared at 70 °C, which was then gradually added to the previously prepared solution and homogenized for another 1 min at 10 000 rpm until a transparent solution was obtained. A blank control was included by replacing the anthocyanin extract with ethanol. These microcapsule suspensions were then used for the preparation of the films.

### Synthesis of gelatine with anthocyanin microcapsules

The gelatine films were prepared by the solution casting method as reported previously (*24*). The gelatine powder (Sigma-Aldrich, Merck) was dissolved in distilled water at 40 °C to form a 5.0% (*m*/*V*) gelatine solution. Then, 3% (*m*/*V*) sorbitol (Merck, Darmstadt, Germany) was added as a plasticizer. The mixture was stirred continuously for 20 min at 100 °C. Then, the anthocyanin microcapsules were added slowly into the solution and the mixture was stirred for 20 min at room temperature to obtain a film-forming solution. The film-forming solution (12 mL) was transferred to a polystyrene Petri dish (10 cm×10 cm) and then placed at 35 °C until completely dried. A control gelatine film was prepared with the same method by replacing the anthocyanin microcapsules with ethanol as a blank control.

### Characterization of the gelatine film colour parameters

The film colour values were determined with a colorimeter (Minolta, Tokyo, Japan) under constant light conditions. The value for L*a*b* were averaged from five measurements of each sample. The total difference in the colour of the films (∆*E*) was calculated according to the following equation:

∆*E*=[(∆*L*)2+(∆*a*)2+(∆*b*)2]·0.5 /1/

where ∆*L*, ∆*a* and ∆*b* are the differences between the colour of the standard colour plate and film samples.

### Scanning electronic microscopy

The microscopic surface characteristics of the films were investigated by scanning electron microscope (Carl Zeiss SMT, Cambridge, UK). Each sample was carefully mounted into grids of dimension 3 mm×0.5 mm and coated with platinum prior to examination.

### Film thickness

The film thickness was measured with an electronic digital micrometer (Mitutoyo Corporation, Kanagawa, Japan). Measurements were replicated six times for each film.

### *Analysis* of *mechanical properties of the films*

The mechanical properties of the films, such as tensile strength (TS), Young's modulus (YM), and elongation at break (E) were analysed according to ASTM standard method D882-10 (*25*). Samples of 2.0 cm×10.0 cm were prepared. The tests were performed using LLOYD LR30K Plus material testing machine (AMETEK Measurement and Calibration Technologies, West Sussex, UK) operated at initial grip separation and crosshead speed set at 50 mm and 50 mm/min, respectively. Five samples were tested for each film, and the average values were presented. Durometer hardness test was done to measure the resistance of the samples to deformation induced by mechanical indentation or abrasion. The control and the film incorporated with anthocyanin microcapsules were subjected to stress using a durometer model M202 (Durotech, London, UK). Shore A hardness measurement was used on the 6 mm thick samples. Five measurements were carried out for each type of film.

### Moisture content of film samples

The film samples of the size 2 cm×2 cm were placed in an aluminium dish and dried in a hot air oven at 90 °C for 24 h (*24*). The initial and final mass of the films were measured. The moisture content was calculated according to the following equation:

*w*(moisture)=[(*m*_i_-*m*_f_)/(*m*_i_)]·100 /2/

where *m*_i_ is the initial mass of the film (g), and *m*_f_ is the mass of the dried film (g).

### Water vapour permeability

Water vapour permeability (WVP) was determined gravimetrically according to the ASTM standard method E96/E96M-16 (*26*). The films were placed on the container with distilled deionized water in a chamber with controlled temperature and relative humidity (23 °C and 15% RH). To determine mass loss, each container covered with the film was weighed immediately and at 1 h intervals for 8 h. The WVP of the films in g/(m^2^∙Pa·s) was calculated according to the following equation:

WVP=(WVTR·*L*)/∆*p* /3/

where WVTR is measured water vapour transmission rate (g/(m^2^·s)) through the film, *L* is mean thickness of the film (m), and ∆*p* is partial water vapour pressure difference (Pa) across the film. For each measurement, at least five repetitions were made.

### Thermal properties

The thermal properties of the films were determined based on the ASTME standard E1131-20 (*27*) and ISO 11358-1:2014 (*28*). Thermogravimetric analysis (TGA) was performed on a Q-5000IR (TA Instruments, New Castle, DE, USA) coupled with a differential thermal analyser (DTA). Each film sample (approx. 5 mg) was heated from 30 to 600 °C with a heating rate of 20 °C/min under nitrogen flow (40 mL/min). The melting point (*T*_m_) was recorded at the temperature at which the endothermic peak appears in the thermograms, and three samples were measured for each film type.

### Fourier-transform infrared spectroscopy

Fourier-transform infrared (FTIR) spectra of the gelatine powder, gelatine control film, anthocyanin extract, and gelatine/anthocyanin microcapsule film were analysed using FTIR spectrometer (Nicolet iS10; Thermo Fisher Scientific, Waltham, MA, USA) operated at a resolution of 4 cm^-1^. The spectrum of each sample was recorded in the wavelength range of 500-4000 cm^-1^.

### Antimicrobial assay against foodborne pathogens

#### Test microorganisms

Three Gram-positive and six Gram-negative foodborne pathogenic bacteria were used to evaluate the antimicrobial effectiveness of films developed in this study: *Staphylococcus aureus*, *Bacillus subtilis*, MRSA, *Escherichia coli*, *Proteus mirabilis*, *Yersinia enterocolitica*, *Salmonella enterica* ser. Typhimurium, *Pseudomonas aeruginosa* and *Acinobacter anitratus*. The test microorganisms were previously isolated from contaminated food samples, verified by their 16S rRNA gene and maintained at Upstream Processing Laboratory, Universiti Kuala Lumpur, Malaysia.

#### Agar diffusion assay

The film antimicrobial properties were determined by the agar diffusion method, as reported previously (*29*) with minor modification. Briefly, the bacterial inoculum was prepared based on the 0.5 McFarland standard (1.5∙10^8^ CFU/mL) and spread on the nutrient agar (Merck, Darmstadt, Germany) as the lawn. Wells of 1 cm in diameter were made on the nutrient agar using agar well borer, and 80 μL of each film solution were loaded into the well and allowed to solidify before incubation at 37 °C. After incubation for 24 h, inhibition zones were determined by measuring the clear zone diameter around the wells.

### Application of the gelatine-anthocyanin films as active packaging for bean curd (tofu)

A mass of 100 g of bean curd (tofu) was cut and wrapped in the control and anthocyanin microcapsule-incorporated gelatine films. The samples were stored for 12 days at two different temperatures, 27 and 4 °C. Sampling was done at an interval of 4 days for microbiological analysis. The bean curd was homogenized in a stomacher (Stomacher 400 Lab System; Seward, Norfolk, UK) with 225 mL of sterile peptone water, followed by serial dilution, and then spread on nutrient agar. After incubation for 24 h at 37 °C, the number of colonies on each plate was counted, and total viable cells (CFU/mg) were calculated.

### Statistical analysis

All tests were repeated at least three times, and data were analysed with SPSS software v. 17.0 (*30*). One-way analysis of variance (ANOVA) was used to evaluate the significance between samples by Duncan's test (p<0.05). Origin v. 8.0 (*31*) and Excel v. 2010 (*32*) were used for statistical analysis.

## RESULTS AND DISCUSSION

**Fig. S1:**
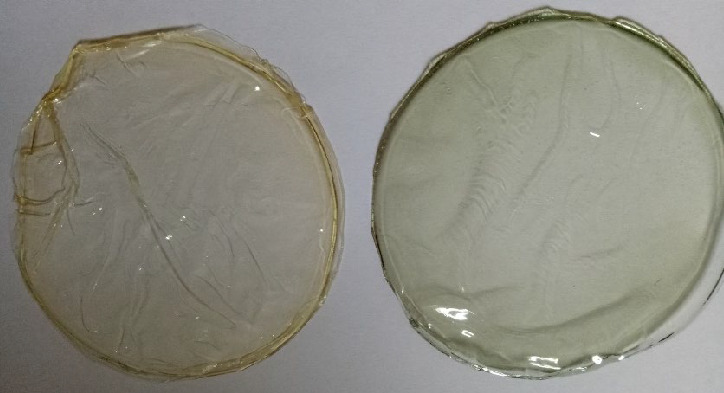
The physical appearance of the gelatine films: control (left) and film with 50 mg of anthocyanin microcapsules (right)

The film total colour difference and surface morphology were observed to study the physiological effect of the incorporated anthocyanin microcapsules. As shown in Table 1, the incorporation of anthocyanin microcapsules in the films increased the blueness (b*) value significantly, while redness (a*), lightness (L*) and ∆*E* values decreased. The obtained results are parallel with the findings of Pourjavaher *et al.* (*33*), where changes of ΔE* value of cellulose nanofibre films were observed after the addition of anthocyanin from red cabbage. The control gelatine film had a thin, yellowish and homogenous surface, while the gelatine film incorporated with anthocyanin microcapsules appeared to be darker and greenish, attributed to the anthocyanin extract colour at pH=6 (Fig. S1). The colour attribute of anthocyanin is a unique feature of this natural pigment. Anthocyanin at different pH will go through a structural transformation associated with colour changes (*34*). In recent years, there has been emerging interest in developing smart packaging material using anthocyanins to detect food spoilage based on the pH changes due to the produced organic acids or ammonia (*35*). In their study in assessing the organoleptic performance of edible flowers, Benvenuti *et al*. (*36*) reported that the anthocyanins in edible flowers significantly improved the sensory profiles and organoleptic performance of food products.

**Table 1:** Physical properties of the gelatine films

Property	Control (Gelatine film)	Gelatine film with anthocyanins
*L*/mm	0.21±0.03	0.31±0.02
TS/MPa	31.0±1.9	37.4±1.1
YM/MPa	607.4±16.3	704.3±11.2
*E*/%	92.8±4.2	102.7±1.6
Hardness/N	28.4±0.7	33.6±1.7
*w*(moisture)/%	12.5±0.6	10.4±0.5
WVP·10^-9^/(g/m^2^·Pa·s)	2.140±0.005	2.840±0.004
L*	45.1±0.2	38.1±0.2
a*	-7.3±0.1	-10.1±0.3
b*	8.11	12.51
∆*E*	31.0 ± 0.2	26.5±0.8

*L*=thickness, TS=tensile strength, YM=Young's modulus, E=elongation at break,, WVP=water vapour permeability, L*=lightness, a*=redness, b*=yellowness, ∆*E*=total colour difference

**Fig. 1:**
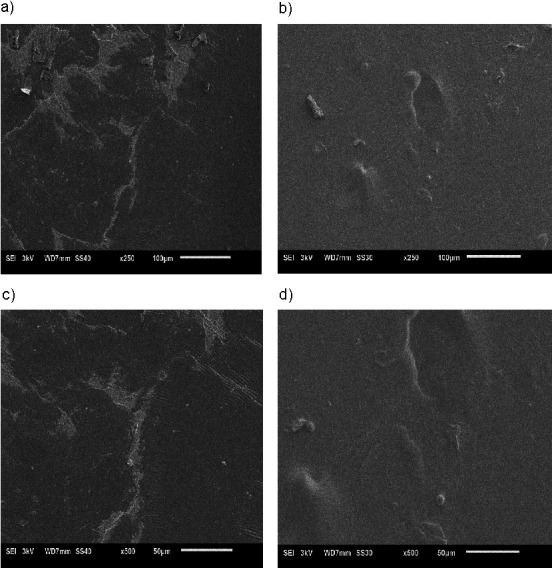
Scanning electron microscopy micrographs showing the surface micromorphology of gelatine films: a) control at 250×, b) film with anthocyanin microcapsules at 250×, c) control at 500× and d) film with anthocyanin microcapsules at 500×

Fig. 1 shows SEM micrographs of the gelatine film surface morphology. A relatively rough surface of the control gelatine film with a substantial presence of pores was observed. However, a smooth and homogeneous surface with fewer pores and cavities was observed on the gelatine film with the incorporated anthocyanin microcapsules. This observation might be due to the differences in the film thickness, which resulted in fewer wrinkles formed on the surface. Rawdkuen *et al*. (*37*) and Wu *et al*. (*38*) reported similar film surface morphology. Grover *et al.* (*39*) reported that the addition of water-soluble carbodiimide during the chemical crosslinking of the gelatine scaffolds resulted in the activation of the carboxylic groups in the gelatine, reducing the number of side reactions and therefore inducing crosslinking with free primary amine groups. Such chemical modification leads to a less porous microstructure, and physicochemical changes of the gelatine scaffolds. Similar pathways may take place during the cross-linking of the gelatine films containing anthocyanin microcapsules.

The mechanical properties of films such as tensile strength (TS) and elongation at break (EAB) are key indicators of the film integrity and resistance to environmental stress during applications such as packaging (*40*). The TS, EAB and Young modulus (YM) of the gelatine films are listed in Table 1. The TS indicates the film strength, while EAB represents the film flexibility prior to breakage. The TS of the control gelatine film was 31.0 MPa, close to the value of mammalian gelatine film reported previously (*41*). With the addition of the anthocyanin microcapsules, the TS increased 20% to 37.4 MPa. The EAB of the control film was 92.8%, while of the gelatine/anthocyanin films it was slightly higher. The produced film was strong but flexible, which is an ideal material for food packaging. Overall, incorporating anthocyanin microcapsules improved the gelatine film mechanical properties, including hardness and YM. The thickness of the gelatine films also increased from 0.2 to 0.3 mm with the addition of the anthocyanin microcapsules. The obtained results are in good agreement with the findings reported by Chin *et al.* (*42*) that antimicrobial agents like *Aloe vera* extract would improve the plasticity and flexibility of gelatine films. The mechanical properties of the films depend on the intermolecular interaction of the film-forming matrix during the establishment of the protein network. Thus, incorporating the anthocyanin microcapsules may increase the hydrogen bonds formed between them and the polypeptides present in the gelatine, which in turn stabilizes the protein network. Furthermore, the heterogeneous film structure might increase the resistance to the film breakage, as previously reported by Libanori *et al.* (*43*). The overall improvement of the gelatine film mechanical properties suggests that anthocyanin microcapsules had a plasticizing effect on the film.

As shown in Table 1, the moisture content of the control film was 12.5% and it decreased to 10.4% by incorporating the anthocyanin microcapsules. In contrast, WVP of the film with anthocyanin microcapsules and control films was 2.84·10^-9^ and 2.14·10^-9^ g/(m^2^·Pa∙s), respectively. Hamilton (*44*) reported that water permeability of low-density polyethylene was 2·10^-8^ g/(m^2^·Pa∙s). The common plastic films have higher water permeability than gelatine films, as gelatine hydrophilicity contributes to the low water barrier properties. However, in the current context, incorporating anthocyanin microcapsules into the gelatine film improved the water permeability and led to better breathable performance of the film.

**Fig. 2:**
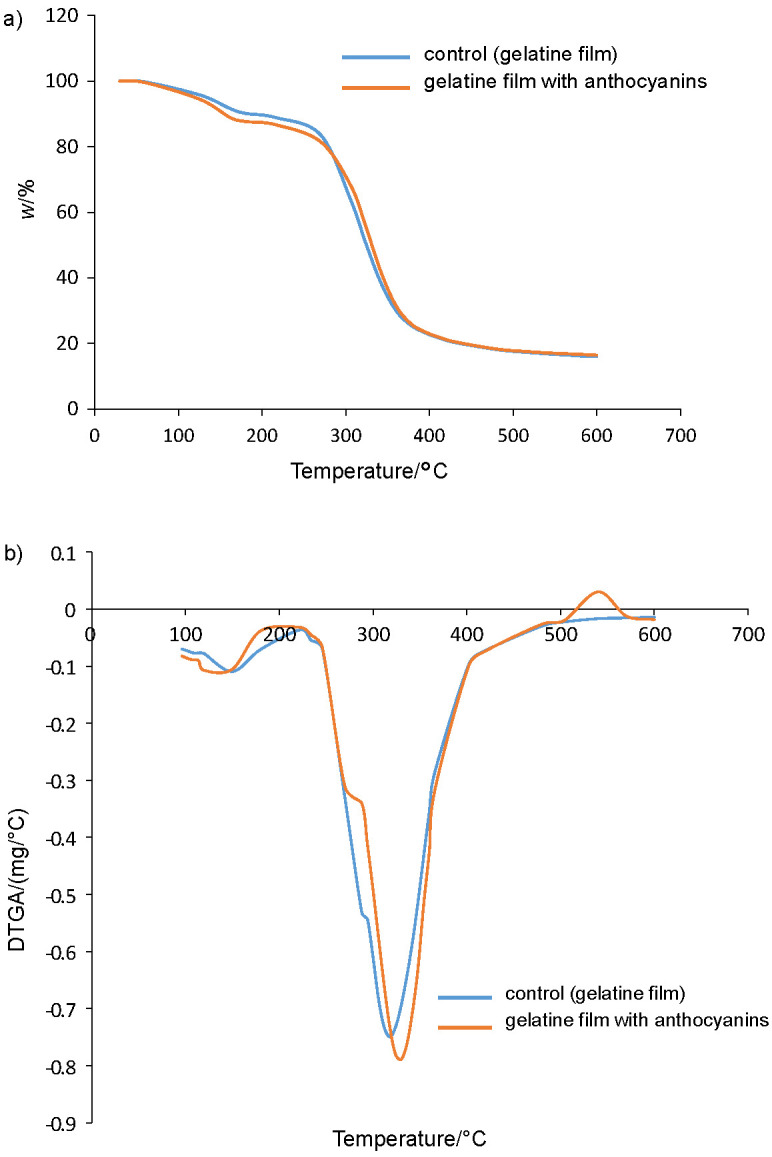
Thermal characterization of gelatine and gelatine/anthocyanin films: a) thermogravimetric analysis curves, and b) derivative thermogravimetry analysis (DTGA)

The thermal properties of the gelatine films are shown in Fig. 2. TGA results show two-step thermal decline patterns for both gelatine and gelatine/anthocyanin films. The first step of the decomposition of both gelatine and gelatine/anthocyanin films started at around 90 °C. The beginning of the second decomposition step was observed at 300 °C due to the evaporation of water remaining in the film, and then the decomposition of gelatine occurred. The temperature of the thermal destruction of gelatine/anthocyanin was slightly delayed compared to the control film. This result indicated that the addition of anthocyanin increased the thermal stability of the film. After the final thermal destruction, the mass fractions of the gelatine and gelatine/anthocyanin film residues were 15.91 and 16.44%, respectively. Mass fraction of gelatine/anthocyanin residue was higher than of gelatine control film due to the presence of anthocyanins in the film. A similar observation was reported by Kanmani and Rhim (*45*) with the addition of AgNPs to the gelatine film.

**Fig. 3:**
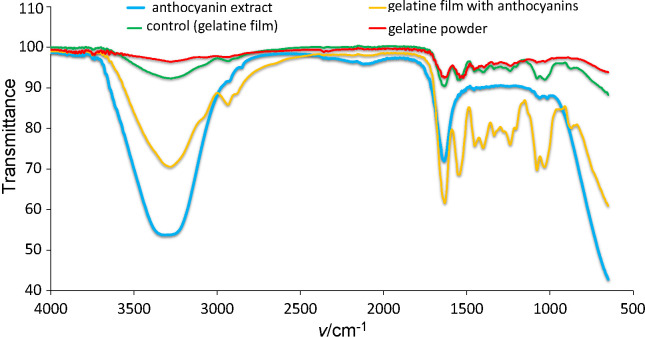
FTIR spectra of anthocyanin extract, gelatine film with anthocyanins, control (gelatine film) and gelatine powder

Fourier-transform infrared (FTIR) spectroscopy was carried out to identify the chemical structure changes of gelatine and the incorporation of anthocyanins. Fig. 3 shows the FTIR spectra of the pure gelatine powder, gelatine (control) film, anthocyanin extracts and gelatine film with incorporated anthocyanins. All spectra show the peak range from 3275 to 665 cm^-1^. The broad and robust absorption peak observed at 3275 cm^-1^ was assigned to hydroxyl strain vibration group (O-H), while the intense peak at 1633 cm^-1^ shows the presence of the carbonyl (C=C) stretching frequency (Table 2). The peaks appearing at 1540 cm^-1^ are due to the stretching vibration of the ester group. The peaks observed in the FTIR spectra of the gelatine powder and gelatine control film show the same bonds and wavenumbers, indicating that there were no major changes in the functional group, whereas the gelatine/anthocyanin film spectra showed a stretching vibration when anthocyanins were added. Hence, it indicates the presence of anthocyanins within the gelatine network of the developed film.

**Table 2:** Comparison of the relevant transmission peaks of gelatine (control) film, gelatine/anthocyanin film, gelatine powder and anthocyanin extract with the standard transmission peak from the literature

Type of bond	Standard transmission peak/cm^-1^	Experimental transmission peak/cm^-1^
C=C of benzene ring	1629.97	1634.36
Carbonyl group	1524.92	1541.53
Aliphatic hydrogen	2934.98	2934.74
OH group	3429	3275.97

The antimicrobial activity of gelatine film incorporated with different anthocyanin microcapsule masses was tested against nine foodborne illness-related microorganisms, including Gram-positive and -negative bacteria. The well diffusion assay shows that 6 out of the 9 test microorganisms are susceptible to the anthocyanin-incorporated gelatine film (Table 3). The developed film exhibited a significant and broad spectrum of antimicrobial activity against both Gram-positive and -negative bacteria, including the common foodborne illnesses caused by bacteria such as *Staphylococcus aureus*, *Escherichia coli*, *Yersinia* sp., *etc*. The broad-spectrum antimicrobial activity of anthocyanins in their free form was also reported by Aly *et al*. (*46*) and Ab Rashid *et al*. (*47*). The size of the inhibition zone increased in a concentration-dependent manner. With the highest concentration of anthocyanins, the inhibition zone size on well diffusion assay varied among all microbes ranging from 10 to 14.2 mm, indicating different susceptibility of the test microorganisms to the anthocyanins. The obtained result agrees with the findings of Werlein *et al*. (*48*) that anthocyanins have better antimicrobial activity against Gram-positive than Gram-negative bacteria. This might occur due to the presence of an outer membrane in the Gram-negative bacteria that acts as a permeability barrier.

**Table 3:** Antibacterial activity of gelatine and gelatine film incorporated with different masses of anthocyanin microcapsules

Test microorganism	*d*(inhibition)/mm						
Control (gelatine film)		*m*(anthocyanins in gelatine)/mg				
		30		40	50	
Gram-positive bacteria							
*Staphylococcus aureus*		-	8.80.3	11.30.7			14.01.0
*Bacillus subtilis*		-	-	-			-
MRSA		-	8.91.8	11.80.4			11.90.1
Gram-negative bacteria							
*Proteus mirabilis*		-	-	-			-
*Yersinia enterocolitica*		-	-	9.30.8			11.50.5
*Escherichia coli*		-	8.30.6	10.30.6			14.21.1
*Salmonella enterica* ser. Typhimurium		-	-	-			-
*Acinobacter anitratus*		-	-	7.90.2			10.01.0
*Pseudomonas aeruginosa*		-	7.60.6	9.01.7			10.01.0

- =no inhibitory zone

**Fig. 4:**
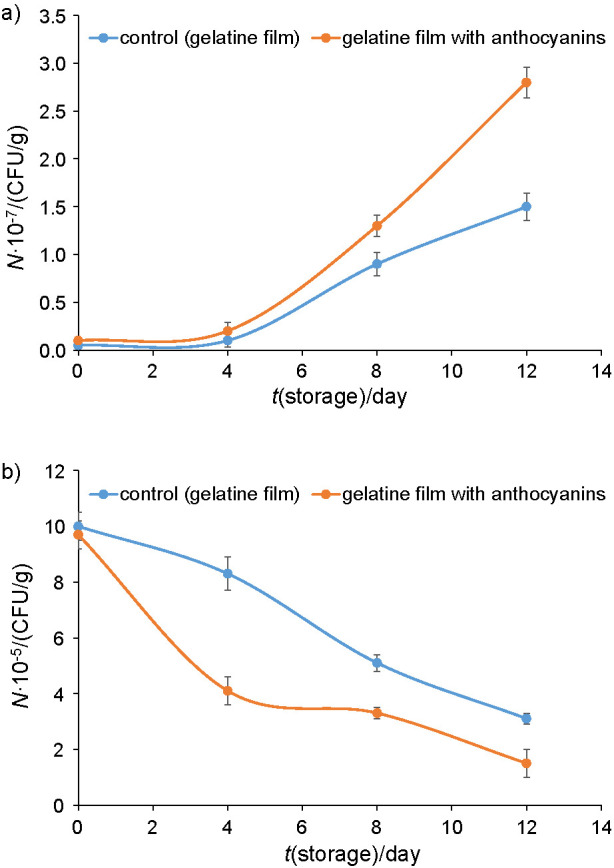
Total viable count changes of the food model with different packaging films during storage for 12 days at: a) room temperature and b) 4 °C

To investigate the efficiency of the developed gelatine film as an active food packaging, we used tofu (soya bean curd) as a food model system. Tofu is a versatile protein-rich food prepared from soya beans. With relatively high moisture content (80–88%), moderate pH (pH=5.8–6.2) and rich protein content (6–8.4%), tofu is a perishable food product with short shelf life (*49*). The detection of spoilage microorganisms such as lactic acid bacteria, *Pseudomonas, Enterobacteriaceae, Bacillus* spp*., Klebsiella* spp. and *Staphylococcus* spp. in tofu have been reported in numerous studies (*49*). Fig. 4 shows total viable count (TVC) of the soya curd using different gelatine films as a packaging system during storage for 14 days at room temperature and 4 °C. The TVC of bacteria is a vital indicator of the quality of food products. At room temperature, the initial TVC was 5.0·10^2^ CFU/g, which suggested that the soya curd quality was low but acceptable. The TVC of the soya curd increased significantly during storage. The TVC of the soya curd packed in the control and gelatine/anthocyanin films was 2·10^3^ and 10^3^ CFU/g, respectively, on day 4, and was close to the maximum microbiological acceptability limit of 10^7^ CFU/g for bean curd. It is suggested that their microbiological shelf life was about 5-6 days. The reported shelf life is longer compared to Yang *et al*. (*50*) where they utilized calcinated shell powder from *Corbicula fluminea* as a natural antimicrobial agent for tofu preservation. No study has been conducted to apply anthocyanins as packing system for tofu. TVC of the samples packed in control gelatine film increased to 1.3·10^4^ and 2.8·10^4^ CFU/g on days 8 and 12, respectively. In contrast, the TVC of the samples packed with gelatine film incorporated with anthocyanin microcapsules was significantly lower than that of the control sample on the same day. The bacterial growth inhibition is particularly significant on day 12 of storage, with the TVC of the control gelatine film two-fold of the film incorporated with anthocyanin microcapsules. These results showed that the sample bacterial growth was inhibited in the packaging system containing gelatine films incorporated with anthocyanin microcapsules. Unlike storage at room temperature, the TVC decreased during 12 days of storage at 4 °C. Nevertheless, the TVC of the samples packed with gelatine film incorporated with anthocyanin microcapsules is continually lower, with significant growth reduction.

## CONCLUSIONS

In conclusion, the incorporation of the anthocyanin microcapsules into the gelatine film significantly improved the mechanical and thermal properties of the film. The developed gelatine film also showed significant bacterial growth inhibition against the common foodborne pathogens. Using soya curd as a food model demonstrates that the developed gelatine film can suppress microbial growth that leads to food spoilage. This study shows that the gelatine film with anthocyanin microcapsules is an effective biodegradable food packaging material with a natural antimicrobial agent that has excellent potential to enhance the shelf life of food products.
